# Surveillance on the endemic of Zika virus infection by meteorological factors in Colombia: a population-based spatial and temporal study

**DOI:** 10.1186/s12879-018-3085-x

**Published:** 2018-04-17

**Authors:** Lung-Chang Chien, Ro-Ting Lin, Yunqi Liao, Francisco S. Sy, Adriana Pérez

**Affiliations:** 1 Department of Environmental and Occupational Health, School of Community Health Sciences, University of Nevada, Las Vegas, 4505 S. Maryland Parkway, Las Vegas, NV 89154 USA; 2 Department of Occupational Safety and Health, China Medical University, No.91 Hsueh-Shih Road, Taichung, 40402 Taiwan; 3 Department of Biostatistics and Data Science, School of Public Health, Houston Campus, The University of Texas Health Science Center at Houston-UTHealth, 1200 Pressler Street, Houston, TX 77030 USA; 4 Department of Biostatistics and Data Science, School of Public Health, Austin Campus, The University of Texas Health Science Center at Houston-UTHealth, 1616 Guadalupe, Suite 6.300, Austin, TX 78071 USA

**Keywords:** Zika virus infection, Meteorological factor, Nonlinear lagged effect, Spatial analysis

## Abstract

**Background:**

Zika virus (ZIKV) infection is a pandemic and a public health emergency. It is transmitted by mosquitoes, primarily the *Aedes* genus. In light of no treatment currently, it is crucial to develop effective vector control programs to prevent the spread of ZIKV infection earlier when observing possible risk factors, such as weather conditions enhancing mosquito breeding and surviving.

**Methods:**

This study collected daily meteorological measurements and weekly ZIKV infectious cases among 32 departments of Colombia from January 2015–December 2016. This study applied the distributed lag nonlinear model to estimate the association between the number of ZIKA virus infection and meteorological measurements, controlling for spatial and temporal variations. We examined at most three meteorological factors with 20 lags in weeks in the model.

**Results:**

Average humidity, total rainfall, and maximum temperature were more predictable of ZIKV infection outbreaks than other meteorological factors. Our models can detect significantly lagged effects of average humidity, total rainfall, and maximum temperature on outbreaks up to 15, 14, and 20 weeks, respectively. The spatial analysis identified 12 departments with a significant threat of ZIKV, and eight of those high-risk departments were located between the Equator and 6°N. The outbreak prediction also performed well in identified high-risk departments.

**Conclusion:**

Our results demonstrate that meteorological factors could be used for predicting ZIKV epidemics. Building an early warning surveillance system is important for preventing ZIKV infection, particularly in endemic areas.

**Electronic supplementary material:**

The online version of this article (10.1186/s12879-018-3085-x) contains supplementary material, which is available to authorized users.

## Background

Zika virus (ZIKV) was first discovered in 1947 from a monkey in the Zika Forest of Uganda. The first human cases of ZIKV infection were reported in 1954 in Nigeria. The first major outbreak of ZIKV occurred in 2007 in the Federated States of Micronesia. The first outbreak in continental South America occurred in 2015 in Brazil, followed by other countries in South America and Asian [1–3]. Now, it has spread to 70 countries with about 1.5 million people infected with ZIKV [4]. Medical research has verified that ZIKV infection is associated with the development of microcephaly in fetuses or babies and Guillian–Barré syndrome in adults [5–7].

ZIKV is a mosquito-borne viral, mainly transmitted by *Aedes aegypti* and *Aedes albopictus* mosquitoes, which also transmit dengue and chikunkunya virus [8]. No cure or vaccine for ZIKV is available so far. Current treatment for ZIKV infection is focused only on treating symptoms like fever, pain, and rash. The development and mass production of ZIKV vaccine might take up to 5 years [9, 10]. While none of the vaccines has been proven to prevent ZIKV infection yet, global and local public health authorities have urged the prevention ZIKV infection by focusing on mosquito control activities and preventing mosquito bite [10, 11].

The transmission of mosquito-borne diseases is associated with weather change [12]. Although *Aedes aegypti* and *Aedes albopictus* mosquitoes have different biting and feeding features, the survival of *Aedes* mosquitoes is highly associated with meteorological factors, such as temperature and humidity [13, 14]. Warmer temperature can shorten the incubation period of *Aedes aegypti*, which might increase the speed of the virus [13]. Previous studies have explored the potential influence of weather change on the abundance of *Aedes* mosquitoes or ZIKV [15, 16]. Hence, the risk of ZIKV transmission is characterized by its sensitivity to weather change.

Previous studies have mapped potential hot spots for the ZIKV transmission [17–19]. Regions with a higher risk of ZIKV epidemic usually had warmer temperature because mosquitoes are more abundant and active [19]. To build an early warning and prevention system, a quantitative measure to capture the risk of new ZIKV epidemic linked to different meteorological factors is needed. However, a full understanding of such dynamic correlations is currently lacking.

With the same transmission as Dengue fever, which has been investigated to have a nonlinear lagged association with weather conditions as well as spatial vulnerability in high-risk areas [20], we supposed that ZIKV infection also had the same scenario. Thus, in this study, we proposed a spatial nonlinear model to investigate the association among ZIKV infection, weather conditions, and lagged effects. We analyzed weekly ZIKV infection cases and meteorological factors among 32 departments in Colombia, from January 2015–December 2016. We hypothesized that a higher risk of ZIKV infection might occur when specific meteorological measurements were observed at most 5 months earlier. The study aims were to: 1) Approximate how early ZIKV infection can be monitored based on meteorological factors; 2) Estimate the highest risk of ZIKV infection behind observed meteorological measurements; 3) Identify high-risk areas of ZIKV infection after controlling for meteorological factors. The ultimate goal of this study was to establish an early warning system for ZIKV infection, which can be flexibly applied in different epidemic areas.

## Methods

### Study area

**Fig. 1:**
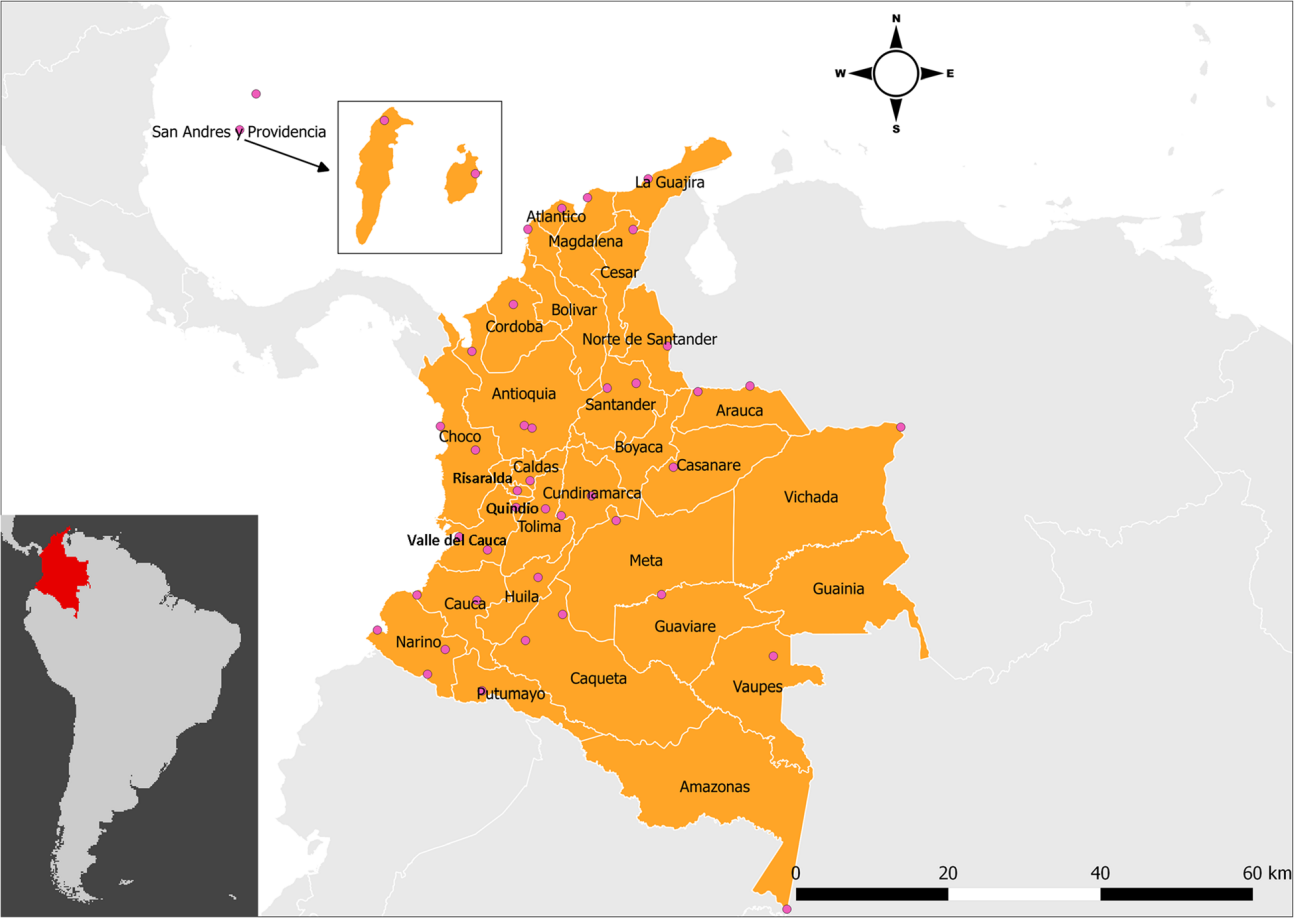
The map of study areas among 32 departments in Colombia, where the pink circles represent the locations of weather monitoring stations

Colombia, the third-most populous country in Latin America is located in the northwest of South America, and has 32 departments (see Fig. 1) [21]. The population of Colombia is concentrated in the Andean highlands and the Caribbean coast. The equator is across the southern Colombia. The climate in Colombia is characterized as tropical, so its warm and isothermal weather condition is a favorable environment for breeding mosquitoes. The Amazon Rainforest, located in the southeastern Colombia, is one of the main habitats of mosquitoes.

### Data source

We collected data on ZIKV infection records and meteorological information in Colombia from 2015 to 2016. These data were collected at the department level, which was analogous to the state level in the United States. Weekly data on ZIKV infection counts of each department were reported to the Colombian National Institute of Health through the public health surveillance system. These data were publicly available at Epidemiological Bulletin in Colombia (http://www.ins.gov.co/buscador-eventos/Paginas/Vista-Boletin-Epidemilogico.aspx). Note that Bogota, the capital of Colombia, only has available ZIKV infection records in 2015 and the first 2 weeks of 2016. A report mentioned that ZIKV infection in Bogota is not endemic because it is at 2640 m above the sea level, and most cases were immigrant cases from people travelling to Bogota from Cundinamarca [22]. Hence, we combined the data from Bogota and Cundinamarca before the 2nd week of 2016. Meteorological factors were obtained from 42 monitoring stations across 32 departments. These data were retrieved from the Weather Underground (https://www.wunderground.com/).

### Meteorological measurements

Weather conditions were quantified by 15 meteorological measurements from 42 monitoring stations in Colombia (Fig. 1), including temperature (minimum; maximum; average), humidity (minimum; maximum; average), dew point temperature (minimum; maximum; average), sea level pressure (minimum; maximum; average), wind speed (maximum; average), and rainfall. Because the missing data rate was as high as 29.3% among those monitoring stations, we chose to apply kriging technique because spatial autocorrelation (measured by Moran’s I) is strong, spatial stratified heterogeneity is weak (measured by q-statistic [23]), and Pearson’s correlation coefficient is small. Evidence in details can refer to Additional file 1: Table S1, Table S2 and Figure S1. Therefore, we especially adopted a local time-space kriging method to impute missing data for each department according to collected measurements along with calendar day and geographic information of monitoring stations (i.e., latitude and longitude) [24]. For matching the weekly ZIKV case data, we first selected the weekly maximum values of maximum temperature, maximum dew point temperature, maximum humidity, maximum sea level pressure, and maximum weed speed from monitoring stations in each of the ten departments with multiple monitoring stations. The weekly minimum values of the previous meteorological factors were determined similarly. The weekly average values for the department of all previous indicated meteorological factors were estimated by average across monitoring stations. For the other 19 departments with only one monitoring station, weekly measurements were derived from maximum, minimum, and average values of corresponding meteorological variables per week. For the three departments without monitoring station, we applied the local time-space kriging method again to impute their meteorological measurements from the other departments. Afterward, we applied a geodetector method in those imputed data, which is a technique to preliminarily detect whether covariates are responsible for an outcome measurement [23].

### Statistical model

In order to take both nonlinear lag effects of meteorological measurements and spatial correlation into account simultaneously, we applied the distributed lag nonlinear model (DLNM) for assessing the nonlinear associations among ZIKV infection, weather, and temporal lagged effect [25]. We assumed that the number of ZIKV infection cases at time t in department d, denoted by Y_dt_, followed a Poisson distribution POI(μ_dt_), where E(Y_dt_) = φμ_dt_, and φ is a scale parameter. The DLNM could be regarded as a generalized linear model with additional cross-basis functions, which are interactions constituted by a nonlinear function for a covariate and a nonlinear function for a lag variable; hence, this study established the DLNM in a quasi-Poisson model framework to take possible over-dispersion into account:

$$ \log \left({\mu}_{dt}\ \right)=\upalpha +\sum \mathrm{CB}\left(\mathrm{Weather},\mathrm{lag}\right)+f(t)+{f}_{spat}\ (d)+\mathrm{offset},\kern0.5em t=1,\dots, 104;d=1,\dots, 32, $$

where *α* is the intercept, and the offset term is the logarithm of population in each department. A natural cubic spline *f*(t) with respect to the calendar time for 104 weeks from 2015 to 2016 is a time smoother with seven degrees of freedom (df) to control long-term temporal autoregressive correlations. Weather conditions and lagged effects were analyzed in the cross-basis function CB(Weather, lag), which is an interaction between a b-spline of a meteorological factor and another b-spline of the lag factor. The spatial function *f*_*spat*_(*d*) describes spatial local heterogeneity of ZIKV incidence by applying Markov random fields, which follow a normal distribution with mean $\sum \limits_{d^{\prime}\in {\Omega}_d}{f}_{spat}\left({d}^{\prime}\right)/{N}_d$ and variance ${\sigma}_d^2/{N}_d$[26]. In details, we defined a neighborhood as an adjacent department *d*^′^ sharing a part of boundaries of another department, and a neighborhood set containing all adjacent departments around the department *d* was denoted by Ω_*d*_. The number of adjacent departments in Ω_*d*_ was denoted by *N*_*d*_, and *f*_*spat*_(*d*^′^) is the spatial estimated effect in each adjacent department. Thus, the mean can be regarded as the average neighborhood effect. In addition, ${\sigma}_d^2$ is the original variance in the department *d*, whereas the number of neighbors diluted its spatial variance. The spatial function can evaluate the remaining variation of ZIKV incidence not explained by meteorological factors. Each department can obtain a spatial estimate from the spatial function, which can be interpreted as the logarithm of relative risk (logRR) in ZIKV of a department compared to the average of all departments. In the other words, the exponential spatial estimate can account for the excessive relative risk (RR) of ZIKV incidence in each department over the whole country. Moreover, the spatial function can detect each department as a high-risk area of ZIKV infection after controlling meteorological factors as the 95% confidence interval (CI) of the spatial estimate significantly greater than zero. We adopted a 3-step model selection to choose the best model. In details, the first step is to determine the best model with only one cross-basis function from 14, 16, 18, and 20 lags by comparing the quasi Akaike information criterion (QAIC) among them. The second step is to determine the best cross-basis function with different degrees of freedoms from 3 to 7 in each meteorolgocial measurement. The third step is selecting the best model with one, two, and three cross-basis functions. Eventually, the best model has three cross-basis functions for average humidity, total rainfall, and maximum temperature with 20 lags. The chosen criterion in each step depends on the smallest QAIC (data not shown but available upon request from the authors). Each estimated cross-basis function was transformed into RR, while the reference levels varied in different meteorological factors. Diagnosis of the residuals were conducted via plots of autocorrelations and partial autocorrelations, and distribution of residuals over time.

Sensitivity analyses were performed by increasing the length of lags to 22 and 24 in the final model. We calculated the similarity of estimated cross-basis function with the first 21 lags (including lag 0) by using the RV-coefficient, which is a correlation measurement between two matrices [27]. Like the general correlation coefficient, a higher RV-coefficient close to one reflects more similarity between two matrices with the same dimension. We also applied the analysis of variance to compare the spatial estimates to see whether the change of lag in cross-basis functions could affect the spatial function.

Data management of this study was carried out in SAS V9·3 (SAS Institute Inc., Cary). Data imputation and model fitting were conducted in the R software, version 3·3·3 (R Development Core Team, 2011). All maps were generated in QGIS v2·18·2. Statistical significance was determined by a type I error level of 0·05.

## Results

**Fig. 2:**
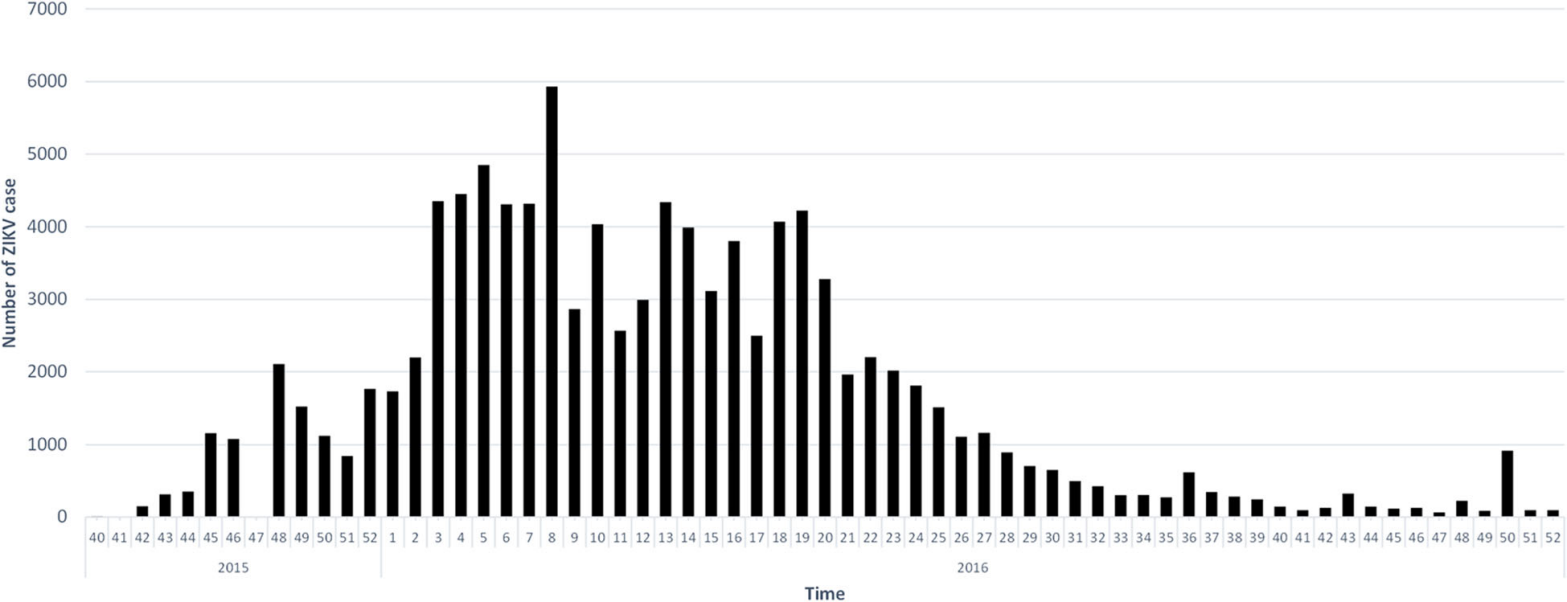
The weekly time trend of ZIKV infection cases in Colombia from the 40th week of 2015 to the 52nd week of 2016

Figure 2 shows the weekly number of ZIKV infection cases since the 40th week of 2015, which is the first week of the official report published by Epidemiological Bulletin in Colombia. Table 1 presents the total ZIKV infection cases and the crude incidence of ZIKV infection among 32 departments in Colombia. Norte de Santander, Santander, and Valle del Cauca had over 10,000 identified ZIKV infection cases from 2015 to 2016, but the three highest crude incidence rates per 10,000 populations were observed in Arauca (120.96), Casanare (140.21), and the islands of San Andres and Providencia (192.37), which were also the only three areas with over 100 ZIKV infection cases per 10,000 populations. The initial covariate detection reveals that most meteorological measurements are significantly responsible for the incidence of ZIKV infection (*p*-values < 0.0001), except for maximum humidity (*p*-value = 0.5288) and maximum wind speed (*p*-value = 0.1193), see Additional file 1: Table S2.

**Table 1:** The total ZIKV infection cases, population, and crude incidence of ZIKV infection among 32 departments in Colombia, 2015 – 2016

Department	# of cases	Weekly cases^a^	Population	Crude incidence (per 10,000 population)
Amazonas	293	2.50	46,950	62.41
Antioquia	2286	19.54	5,601,507	4.08
Arauca	1851	15.82	153,028	120.96
Atlantico	6743	57.63	2,112,001	31.93
Bolivar	1893	16.18	1,836,640	10.31
Boyaca	374	3.20	1,210,982	3.09
Caldas	384	3.28	898,490	4.27
Caqueta	1212	10.36	337,932	35.87
Casanare	3944	33.71	281,294	140.21
Cauca	265	2.26	1,182,022	2.24
Cesar	1596	13.64	878,437	18.17
Choco	22	0.19	388,476	0.57
Cordoba	2931	25.05	1,462,909	20.04
Cundinamarca	5083	43.44	9,007,373	5.64
Guainia	17	0.15	18,797	9.04
Guaviare	195	1.67	56,758	34.36
Huila	6963	59.51	1,001,476	69.53
La Guajira	673	5.75	655,943	10.26
Magdalena	3180	27.18	1,136,819	27.97
Meta	4301	36.76	713,772	60.26
Narino	59	0.50	1,498,234	0.39
Norte de Santander	10,512	89.85	1,208,336	87.00
Putumayo	538	4.60	237,197	22.68
Quindío	397	3.39	518,691	7.65
Risaralda	1385	11.84	859,666	16.11
San Andres and Providencia	1146	9.79	59,573	192.37
Santander	10,069	86.06	1,913,444	52.62
Sucre	1843	15.75	762,263	24.18
Tolima	7079	60.50	1,312,304	53.94
Valle del Cauca	26,908	229.98	4,052,535	66.40
Vaupes	0	0.00	19,943	0.00
Vichada	41	0.35	44,592	9.19

^a^The weekly cases started from the 40th week of 2015, which was the first report released by Epidemiological Bulletin in Colombia

**Fig. 3:**
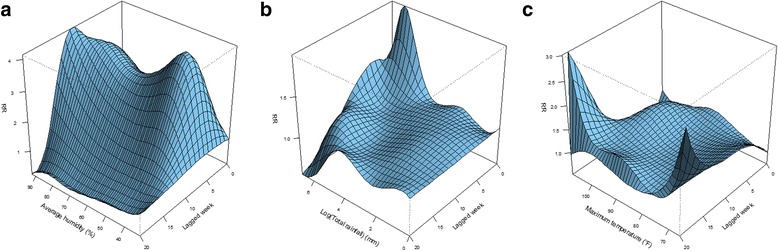
Relative risk (RR) of Zika virus (ZIKV) infection along lagged weeks and three selected meteorological measurements in terms of (**a**) average humidity, (**b**) logarithm of total rainfall, and (**c**) maximum temperature

**Fig. 4:**
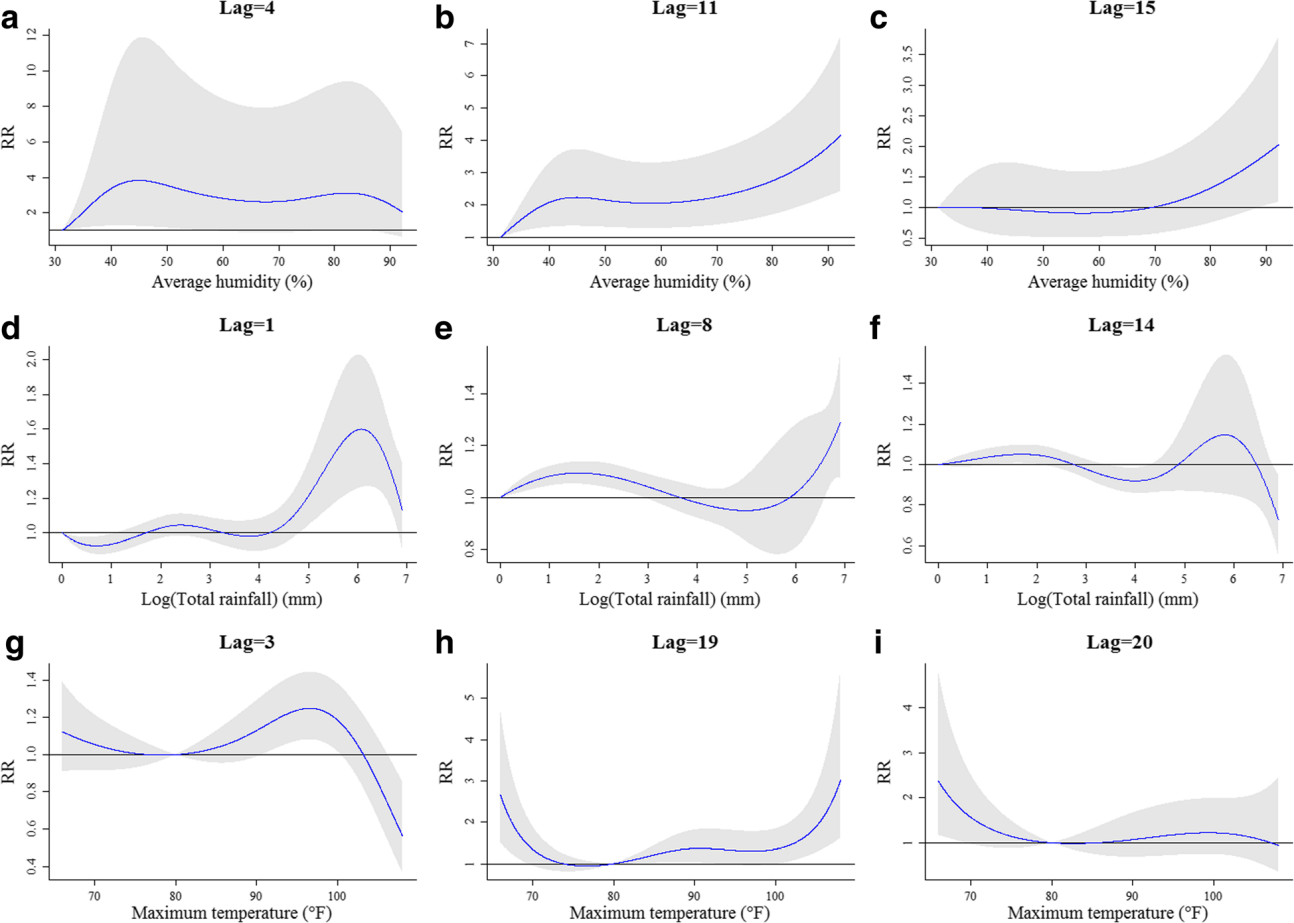
Relative risk (RR) of Zika virus (ZIKV) infection at selected lags in three selected meteorological measurements. In average humidity, the selected lags are (**a**) 4 weeks, (**b**) 11 weeks, and (**c**) 15 weeks. In logarithm of total rainfall, the selected lags are (**d**) 1 week, (**e**) 8 weeks, and (**f**) 14 weeks. In maximum temperature, the selected lags are (**g**) 3 weeks, (**h**) 19 weeks, and (**i**) 20 weeks

Defining the lowest average humidity as the reference level by 31·29%, Fig. 3a shows a great surf around the top and right corners, indicating a large RR of ZIKV infection increased across all average humidity levels within 10 lagged weeks. The outbreak of ZIKV infection can be observed at least after 4 weeks (Fig. 4a), and up to 15 weeks (Fig. 4c) based on significant RRs. Longer lagged weeks with a large RR can be detected more likely in higher average humidity. Table 2 shows that the estimated cross-basis function of average humidity can detect the largest significant RR by 4·16 (95% CI = 2·41, 7·19) at lag 11 when the average humidity reached 92·14%. The whole variation of RR at lag 11 in Fig. 4b reveals that all levels of average humidity had the RR of ZIKV infection significantly greater than one after 11 weeks.

**Table 2:** The largest relative risk of ZIKV infection in average humidity, total rainfall, and maximum temperature by lagged week

Lag (Week)	Average humidity (%)	Largest RR	95% Confidence interval	Logarithm of total rainfall (mm)	Largest RR	95% Confidence interval	Maximum temperature (°F)	Largest RR	95% Confidence interval
0	84.91	1.98	(0.33, 11.86)	5.99	1.98	(1.33, 2.94)	87.69	1.14	(0.90, 1.45)
1	83.52	2.25	(0.48, 10.62)	6.08	1.60	(1.26, 2.02)	90.15	1.13	(0.94, 1.36)
2	44.44	2.82	(0.70, 11.37)	6.34	1.38	(1.19, 1.59)	94.90	1.18	(1.01, 1.37)
3	44.74	3.40	(0.97, 11.90)	6.91	1.38	(1.13, 1.69)	96.56	1.25	(1.08, 1.44)
4	44.94	3.83	(1.24, 11.84)	6.91	1.44	(1.17, 1.78)	97.24	1.31	(1.13, 1.53)
5	45.07	4.06	(1.48, 11.16)	6.91	1.46	(1.18, 1.80)	97.66	1.36	(1.16, 1.58)
6	45.16	4.07	(1.66, 9.98)	6.91	1.43	(1.17, 1.75)	97.99	1.38	(1.18, 1.61)
7	45.22	3.88	(1.77, 8.52)	6.91	1.37	(1.13, 1.66)	98.28	1.39	(1.20, 1.61)
8	87.77	3.70	(1.88, 7.28)	6.91	1.29	(1.07, 1.55)	98.58	1.39	(1.20, 1.60)
9	92.14	3.92	(2.08, 7.37)	6.91	1.19	(0.99, 1.43)	66.00	1.38	(1.14, 1.67)
10	92.14	4.14	(2.34, 7.33)	6.61	1.10	(0.95, 1.27)	66.00	1.39	(1.15, 1.69)
11	92.14	4.16	(2.41, 7.19)	6.15	1.10	(0.87, 1.39)	66.00	1.40	(1.12, 1.76)
12	92.14	3.93	(2.25, 6.86)	5.99	1.12	(0.87, 1.46)	66.00	1.43	(1.09, 1.86)
13	92.14	3.44	(1.91, 6.19)	5.89	1.14	(0.86, 1.51)	66.00	1.47	(1.08, 1.98)
14	92.14	2.77	(1.50, 5.11)	5.82	1.15	(0.85, 1.54)	108.00	1.55	(1.03, 2.32)
15	92.14	2.03	(1.09, 3.77)	5.74	1.13	(0.84, 1.52)	108.00	1.84	(1.24, 2.72)
16	92.14	1.33	(0.73, 2.44)	5.62	1.10	(0.83, 1.45)	108.00	2.15	(1.48, 3.13)
17	31.29	1.00	(1.00, 1.00)	5.41	1.04	(0.82, 1.33)	108.00	2.47	(1.70, 3.60)
18	31.29	1.00	(1.00, 1.00)	0.53	1.02	(0.98, 1.05)	108.00	2.77	(1.78, 4.32)
19	31.29	1.00	(1.00, 1.00)	4.19	1.05	(0.95, 1.16)	108.00	3.01	(1.61, 5.63)
20	31.29	1.00	(1.00, 1.00)	4.09	1.12	(0.98, 1.28)	66.00	2.37	(1.17, 4.78)

Figure 3b presents a peak in the top corner, indicating that a higher RR may more likely happen in a shorter lag and a large amount of rainfall. Defining no rainfall as the reference level, besides present week (lag 0), the most significant outbreak of ZIKV infection (RR = 1·60; 95% CI = 1·26, 2·02) can be observed after 1 week, when the weekly total rainfall accumulated to 437·03 mm (i.e., e^6·08^), see Table 2. Similar as average humidity, higher total rainfall was more related to ZIKV infection outbreak. However, we also observed some significant lagged effects in both high and low total rainfall. For instance, Fig. 4e reveals that the largest RR of ZIKV infection at lag 8 was 1·29 (95% CI = 1·07, 1·55) under an extreme rainfall over 1000 mm. The significant lag effect can last up to lag 14 when the weekly total rainfall increased at least 3·10 mm (i.e., e^1·13^), see Fig. 4f.

As the reference level was defined at 80 °F, the variation of RR computed from the estimated cross-basis function of maximum temperature reveals two peaks (Fig. 3c), demonstrating that a longer lag effect may happen in both lower and higher maximum temperatures. The shortest lagged effect was observed at lag 3 when the maximum temperature was between 90·61 °F and 100·43 °F (i.e., 32·56 °C and 38·02 °C), see Fig. 4g. Table 2 reveals that the largest RR (3·01; 95% CI = 1·61, 5·63) was observed at lag 19 when the maximum temperature increased to 108·00 °F (i.e., 42·22 °C). The longest lagged effect can be detected at lag 20 when the maximum temperature was lower than 69·38 °F, see Fig. 4i.

**Fig. 5:**
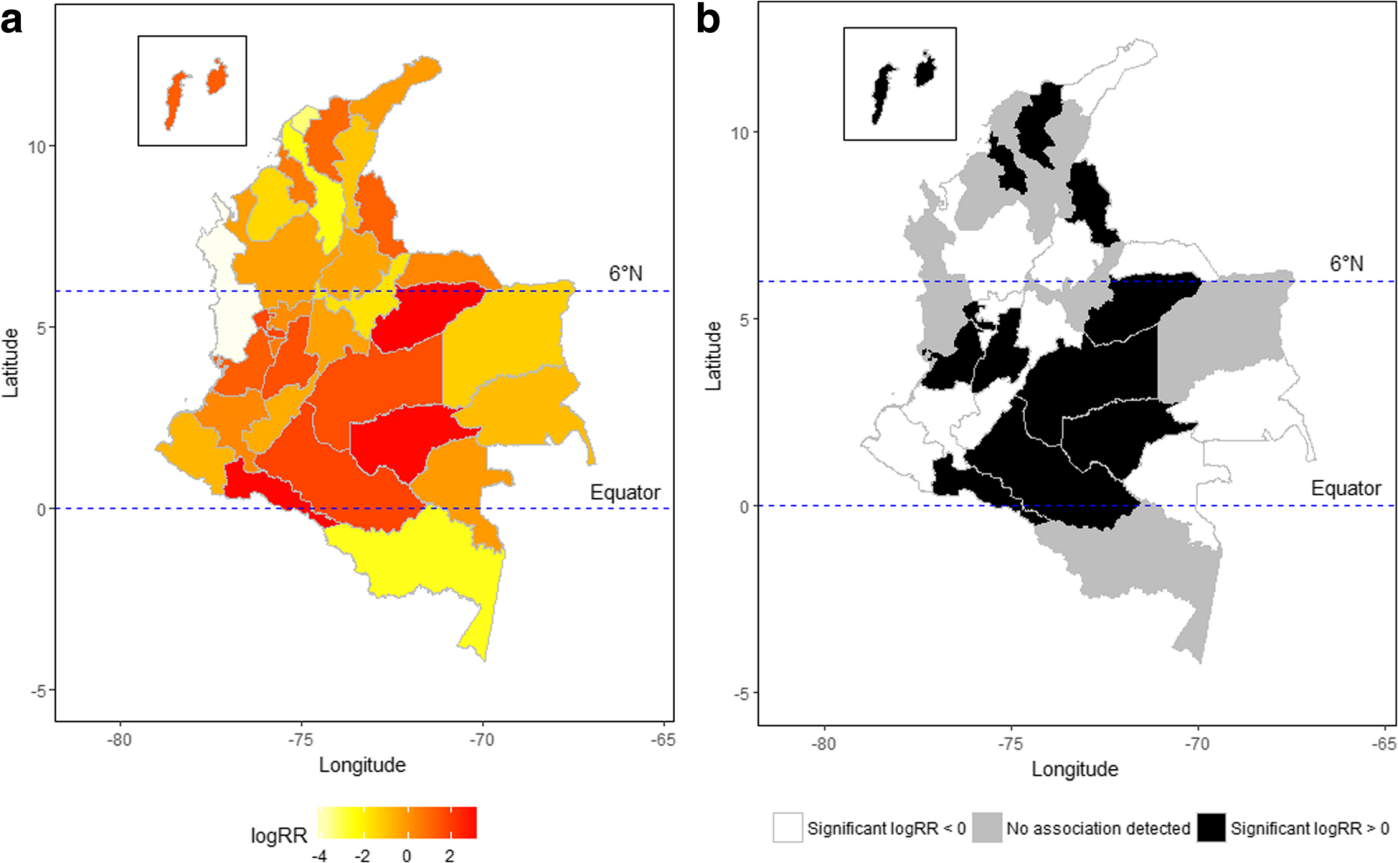
Geospatial pattern of Zika virus (ZIKV) infection among 32 departments in Colombia during 2015–2016 in terms of (**a**) spatial estimate and (**b**) spatial significance

Figure 5 depicts the map from the estimated spatial function, revealing a larger logRR located in the middle or south Colombia. Seventeen of the 32 departments were positively associated with ZIKV infection, but only 12 of them can be defined as ZIKV high-risk areas because of a 95% CI strictly greater than zero. The result indicates that, after controlling for the meteorological effects, 37·50% (12 out of 32) of departments still showed a significantly higher risk for ZIKV infection. These departments are more likely located closer to the equator. More specifically, if Colombia was split by latitude at 6°N, only four high-risk departments (Sucre, Magdalena, Norte de Santander, and San Andres) were located above 6°N, and the other eight high-risk departments were all located between the equator and 6°N. The significantly lowest risky area was Sucre (logRR = 0·53, 95% CI = 0·07, 0·99), which is the northeast high-risk department in the mainland territory of Colombia. The significantly highest risky area was Casanare (logRR = 3·34; 95% CI = 2·91, 3·76). The equator was across three high-risk departments, including Putumayo, Caqueta, and Guaviare.

We compared the results of lag 0–20 weeks with those at lag 22 and 24 weeks and found similar estimates in the three cross-basis functions (data not shown). The RV-coefficients were all at least 0·93, suggesting that despite using a longer length of lags, the first 21 estimates (i.e., lag 0 to lag 20) were still similar to the original results from 20 lags. Moreover, no obvious change was found in the estimated spatial function when considering more lags (all *p*-values > 0·99). Compared to the final model with 20 lags, the mean square errors were 0·06 and 0·12 when using lag 22 and 24 weeks in the DLNM, respectively. The change of lag period did not affect the identification of high-risk areas. The spatial estimates kept the same order among these departments.

## Discussion

This study established an early warning model to project the outbreak of ZIKV infection when a weather condition with a certain measurement was observed up to 20 weeks ago. We proposed a modeling selective process to determine which meteorological factors should be used. The approach can be applied flexibly in other countries with high prevalence of ZIKV infections, where meteorological measurements are available. Our findings suggest that, averaged humidity, total rainfall, and the maximum temperature can adequately detect a ZIKV infection outbreak at least 3 months in advance. Since the proposed model can examine significantly positive lagged effects and geographic impacts, our findings will be applicable in the development of a ZIKV surveillance system by governmental agencies.

The lagged effect of weather change on ZIKV infection has not been well investigated previously. The traditional approach of using internet search, social media, and news reports can predict ZIKV infection outbreaks at most 3-week ahead of time [28]. This short prediction may not provide sufficient time to implement intervention programs to prevent ZIKV infection outbreak, especially in rural areas. Dengue fever has a similar transmission mechanism as Zika fever, was observed to have varied and longer lagged effects in Vietnam (9–12 weeks), Singapore (3 months), and Taiwan (15–18 weeks) [20, 29, 30]. These studies all highlighted that: (1) longer prediction time could provide adequate time for governmental agencies to respond; (2) meteorological factors are the main predictors of the disease outbreak, and they are easily accessible and manageable. Hence, using meteorological factors to monitor ZIKV infection outbreaks is reasonable, and it provides an earlier warning because ZIKV and dengue virus have the same mode of transmission pathway by mosquito vectors.

The significant association between ZIKV infection and meteorological factors is expected because vector-borne diseases are highly correlated with weather conditions, while advanced scientific evidence still needs further investigation besides this study. A study in Brazil verified a more direct linkage between daily rainfall, humidity, and mean temperature and ZIKV infection with human incubation and infectious periods of 4·8 days and 3·6 days, respectively [31]. Rainfall, humidity, and temperature were also used in developing a risk assessment model for calculating the ZIKV risk index [32].

Some of the significant high-risk departments identified by the spatial function in Colombia were separately investigated in previous studies. The earliest research in Colombia estimated the basic reproductive number for the ZIKV outbreak in San Andres and Girardot (in the department of Cundinamarca) using data from September 2015 to January 2016 [33]. The estimated cumulative incidence rates of ZIKV infection in over a half of municipalities in Sucre and Tolima showed a high incidence rate of over 100 cases per 100,000 population [34, 35]. We further verified that all these areas were at high risk for ZIKV infection after controlling for weather conditions.

This study is the first epidemiological research covering a two-year period among several epidemic areas of ZIKV in Colombia. We conducted a population-based study to examine significant associations between ZIKV infection risk and weather change. Because there is currently no curative treatment and vaccine for ZIKV infection, preventing mosquito bites at individual level and vector control measures at local, regional, and national level remain the high priority for disease prevention and control. If we can estimate a potential hazard of ZIKV infection earlier by using existing measurements, there will be more time to prepare and develop effective prevention and control programs for vector-borne diseases. Building a surveillance system that can investigate the occurrence of ZIKV infection earlier will be useful in strengthening the efficiency of vector control programs. In addition, using meteorological measurements in a surveillance system is convenient and fast because they are usually measured at monitoring stations hourly and daily. Our research findings may not exactly reflect the same scenario in other areas, such as Brazil or Puerto Rico, because the result of the data analysis may be affected by the completeness of data, the length of the study period, and the geographic location of the study area. However, this study proposed a systematic approach to conduct the surveillance system, which can be modified easily and flexibly in the other areas.

Using the DLNM for monitoring ZIKV infection by meteorological factors provides a systematic method for building an early warning system. The nonlinear lag analysis and spatial analysis might be applied separately, while a study has proven the importance of including a spatial function in the DLNM [36], especially in controlling possible overestimation when analyzing spatiotemporally imputed data. Practically, this approach can be adopted in any epidemic area when time series data are available. Since ZIKV has been verified to cause microcephaly in neonates [37], pregnant women living in high-risk areas should be alarmed in advance. Microcephaly can be detected as early as 18–20 weeks gestation [38]. Therefore, as the outbreak of ZIKV infection can be monitored in advance, the outbreak of microcephaly might be detected earlier.

There are some limitations in this study. First, the study period was still too short because the first official report of ZIKV infection cases was not released until the 40th week of 2015 in Colombia. We believe that, like dengue fever, there should be a seasonal variation in Zika fever, while it was unlikely to have better control in the statistical model with only 2-year data. Second, the Zika fever might be under-reported, especially in the beginning of October in 2015. During that period, all departments might not have developed a systematic process to diagnose and report ZIKV infection. Third, the reported ZIKV infection data did not differentiate between those acquired locally and those acquired from other countries. We were also unable to verify whether a case was caused by mosquito bites or transmitted by sexual activities. Fourth, the interaction term between the nonlinear smoothing function of time and the spatial function is unavailable in the model, so a real spatiotemporal pattern was unable to be investigated in this study. Further research will need to incorporate sociodemographic, interpersonal/intrapersonal characteristics to the current model to better understanding other potential factors associated with ZIKV infection. Fourth, measurement bias may exist because the meteorological measurements were collected at the community level rather than the individual level. Lastly, this study may have an uncertainty of causal influence because of ecological design. Conducting individual-based analyses may overcome the two limitations if individual-level data are available.

Future work will rely on data with a longer study period to obtain more accurate findings. Because ZIKV can be spread by infected persons, we need to have a more comprehensive surveillance to investigate whether ZIKV incidence has a significant spatial variation over time. More importantly, further analyses are needed to evaluate whether prevention, which is carried out in a certain period of time suggested by our model, can significantly reduce the number of ZIKV infection, especially in high-risk areas.

## Conclusions

ZIKV is a pandemic and a public health emergency. Unlike other vector-borne diseases, it does not only affects people who are bitten by mosquitoes, but also increase the risk of microcephaly in infants born to infected mothers. Although ZIKV infection and microcephaly are not associated with high mortality, it may cause unpredictable medical cost and family burden. Since we do not have curative treatment and vaccine for ZIKV, it is important to develop an early warning system that is efficient and inexpensive to implement because of better technique of monitoring weather change. We anticipate that the proposed approach in this study can be applied in other highly endemic areas. We believe that the more data collected in the future can help build a better early warning system for ZIKV infection.

## Additional file

Additional file 1: **Table S1.** Moran’s I test statistic of meteorological measurements. **Table S2** Q-statistic for meteorological measurements before and after imputations. **Figure S1** Scatter plots of Zika virus infection cases versus each of 15 meteorological measurements. (DOCX 907 kb)

## Abbreviations

CI: Confidence interval
DLNM: Distributed lag nonlinear model
logRR: Logarithm of relative risk
RR: Relative risk
ZIKV: Zika virus
