# Supplementary material for: Surveillance on the endemic of Zika virus infection by meteorological factors in Colombia: a population-based spatial and temporal study
Source: BMC Infect Dis. 2018 Apr 17;18:180. doi: 10.1186/s12879-018-3085-x (PMC5905128; doi:10.1186/s12879-018-3085-x)

**Appendix**

**Surveillance on the Endemic of Zika Virus Infection by Meteorological Factors in Colombia: A Population-Based Spatial and Temporal Study**

Lung-Chang Chien, Ro-Ting Lin, Yunqi Liao, Francisco S. Sy, Adriana Perez

Table S1. Moran’s I test statistic of meteorological measurements.

| **Meteorological factor** | **Observed index value** | **Expected index value** | **Standard deviation** | **Z** | **P-value** |
| --- | --- | --- | --- | --- | --- |
| Maximum temperature | 0.1585 | -0.000402 | 0.00043 | 369.8 | <.0001 |
| Minimum temperature | 0.1716 | -0.000402 | 0.00043 | 400.2 | <.0001 |
| Average temperature | 0.2174 | -0.000416 | 0.00045 | 485.1 | <.0001 |
| Maximum dew point temperature | 0.1968 | -0.000402 | 0.00043 | 458.9 | <.0001 |
| Minimum dew point temperature | 0.1163 | -0.000402 | 0.00043 | 271.6 | <.0001 |
| Average dew point temperature | 0.2008 | -0.000402 | 0.00043 | 468.1 | <.0001 |
| Maximum humidity | 0.0412 | -0.000402 | 0.00043 | 96.8 | <.0001 |
| Minimum humidity | 0.0541 | -0.000402 | 0.00043 | 126.9 | <.0001 |
| Average humidity | 0.0695 | -0.000402 | 0.00043 | 162.7 | <.0001 |
| Maximum sea level pressure | 0.1596 | -0.000397 | 0.00042 | 378 | <.0001 |
| Minimum sea level pressure | 0.1273 | -0.000397 | 0.00042 | 301.7 | <.0001 |
| Average sea level pressure | 0.2075 | -0.000397 | 0.00042 | 491.1 | <.0001 |
| Maximum wind speed | 0.0101 | -0.000398 | 0.00042 | 24.8 | <.0001 |
| Average wind speed | 0.1313 | -0.000398 | 0.00042 | 311 | <.0001 |
| Rainfall | 0.0666 | -0.000396 | 0.00042 | 158.9 | <.0001 |

Table S2. Q-statistic for meteorological measurements before and after imputations.

|  | **Before imputation** | | **After imputation** | |
| --- | --- | --- | --- | --- |
| **Meteorological factor** | **q-statistic** | **p-value** | **q-statistic** | **p-value** |
| Maximum temperature | 0.0060 | <.0001 | 0.0074 | <.0001 |
| Minimum temperature | 0.0157 | <.0001 | 0.0125 | <.0001 |
| Average temperature | 0.0160 | <.0001 | 0.0074 | <.0001 |
| Maximum dew point temperature | 0.0184 | <.0001 | 0.0127 | <.0001 |
| Minimum dew point temperature | 0.0110 | <.0001 | 0.0074 | <.0001 |
| Average dew point temperature | 0.0135 | <.0001 | 0.0121 | <.0001 |
| Maximum humidity | 0.0028 | 0.0092 | 0.0004 | 0.5288 |
| Minimum humidity | 0.0081 | <.0001 | 0.0092 | <.0001 |
| Average humidity | 0.0065 | <.0001 | 0.0105 | <.0001 |
| Maximum sea level pressure | 0.0173 | <.0001 | 0.0129 | <.0001 |
| Minimum sea level pressure | 0.0140 | <.0001 | 0.0134 | <.0001 |
| Average sea level pressure | 0.0150 | <.0001 | 0.0161 | <.0001 |
| Maximum wind speed | 0.0039 | 0.0254 | 0.0022 | 0.1193 |
| Average wind speed | 0.0038 | 0.0264 | 0.0036 | 0.0179 |
| Rainfall | 0.0063 | <.0001 | 0.0031 | 0.0336 |

Figure S1. Scatter plots of Zika virus infection cases versus each of 15 meteorological measurements.


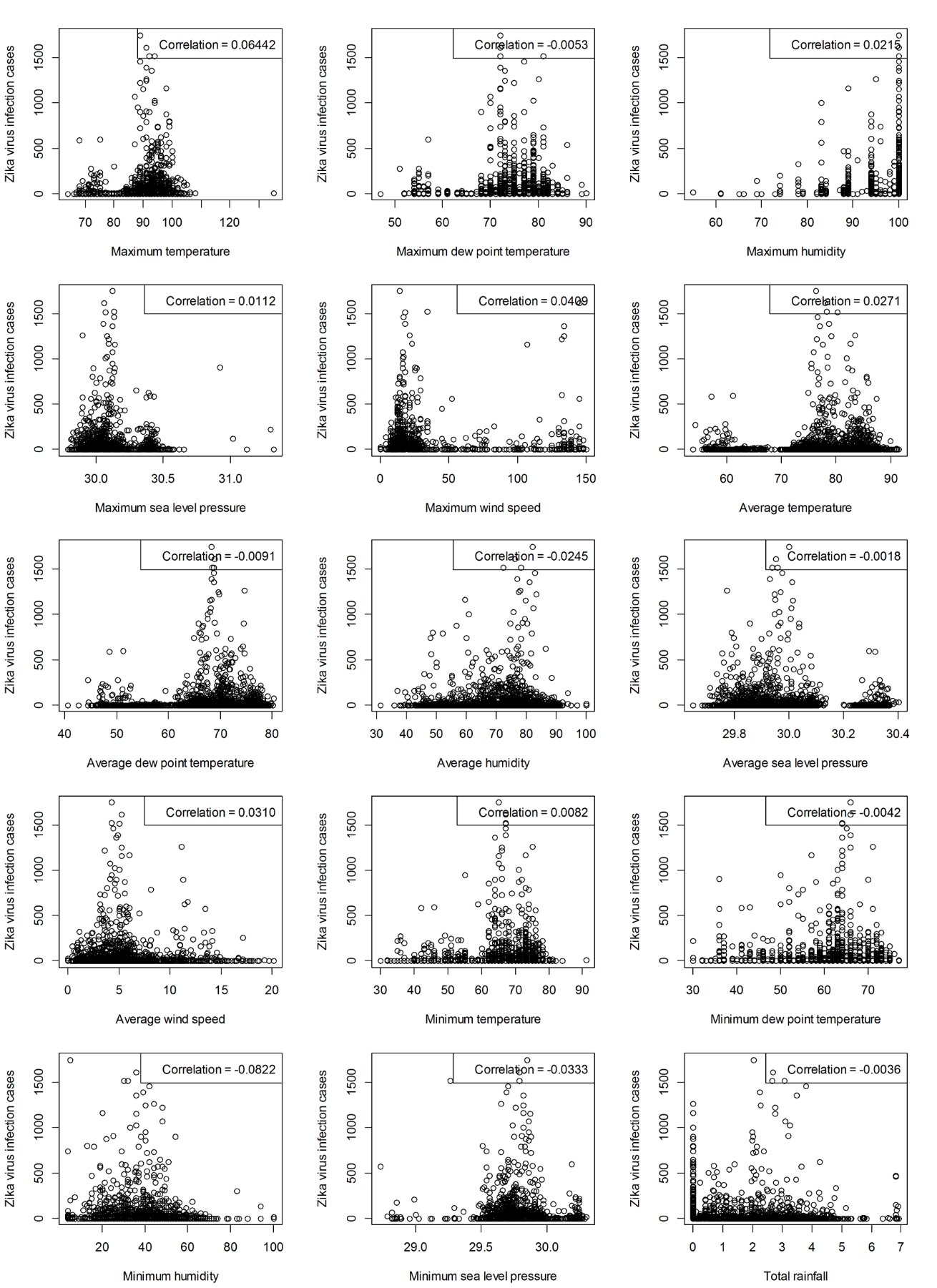

Supplement: Supplementary file 1 — Table S1. Moran’s I test statistic of meteorological measurements. Table S2 Q-statistic for meteorological measurements before and after imputations. Figure S1 Scatter plots of Zika virus infection cases versus each of 15 meteorological measurements. (DOCX 907 kb) [file 12879_2018_3085_MOESM1_ESM.docx]
